# Incorporating historical control data into the design and analysis of clinical trials

**DOI:** 10.1186/1745-6215-16-S2-P205

**Published:** 2015-11-16

**Authors:** Maxine Bennett, William Powley, Jeffrey Wetherington, Adrian Mander

**Affiliations:** 1 MRC Biostatistics Unit, Cambridge, UK; 2 GlaxoSmithKline, Stevenage, UK; 3 GlaxoSmithKline, Pennsylvania, USA

## 

A standard two-arm randomised controlled trial usually compares a novel treatment to a standard of care. Patients are randomised 1:1 to each treatment and the analysis is based only on patients within the current trial. While historical data is often used informally to design trials, in choosing endpoints and forming the basis of sample size calculations, only recently has it been considered for formal use in the design and analysis of trials.

Often, one or more trials have been conducted on the standard of care. Incorporating historical control data could potentially lead to more efficient trials. This could allow more patients to be randomised to the novel treatment, reduce the sample size and decrease the study duration when historical and current data are commensurate. However, when the data are inconsistent, there is a potential for bias, inflated type I error and reduced power.

We propose an adaptive design. An interim analysis is performed to assess the historical-current data conflict. The randomisation ratio is adapted to allocate more patients to the novel treatment and less to control if there is no conflict. The final analysis uses a weighted combination of the historical and current control data. Two weights are proposed, the percentage of overlap in the two control distributions and a weight based on tail area probabilities. This design increases power when there is no conflict and reverts back to a standard design when the conflict is too great. These methods are illustrated with an example and the operating characteristics are compared to those of historical data methods proposed in the literature.

